# Mixed methods process evaluation of my breathing matters, a digital intervention to support self-management of asthma

**DOI:** 10.1038/s41533-021-00248-6

**Published:** 2021-06-04

**Authors:** Kate Greenwell, Ben Ainsworth, Anne Bruton, Elizabeth Murray, Daniel Russell, Mike Thomas, Lucy Yardley

**Affiliations:** 1 Centre for Clinical and Community Applications of Health Psychology, School of Psychology, Faculty of Environmental and Life Sciences, University of Southampton, Southampton, UK; 2 Department of Psychology, Faculty of Humanities and Social Sciences, University of Bath, Bath, UK; 3 Respiratory Biomedical Research Unit, University Hospitals Southampton NHS Foundation Trust, Southampton, UK; 4 School of Health Sciences, Faculty of Environmental and Life Sciences, University of Southampton, Southampton, UK; 5 Primary Care and Population Health, University College London, London, UK; 6 Patient Contributor, Southampton, UK; 7 Primary Care, Population Sciences and Medical Education (PPM), University of Southampton, Southampton, UK; 8 School of Psychological Science, University of Bristol, Bristol, UK

**Keywords:** Asthma, Patient education

## Abstract

This study aimed to explore user engagement with ‘My Breathing Matters’, a digital self-management intervention for asthma, and identify factors that may influence engagement. In a mixed methods design, adults with asthma allocated to the intervention arm of a feasibility trial (*n* = 44) participated in semi-structured interviews (*n* = 18) and a satisfaction questionnaire (*n* = 36) to explore their views and experiences of the intervention. Usage data highlighted that key intervention content was delivered to most users. The majority of questionnaire respondents (78%; *n* = 28) reported they would recommend the intervention to friends and family. Interviewees expressed positive views of the intervention and experienced several benefits, mainly improved asthma control, medication use, and breathing technique. Factors that may influence user engagement were identified, including perceptions of asthma control, current self-management practices, and appeal of the target behaviours and behaviour change techniques. Findings suggested My Breathing Matters was acceptable and engaging to participants, and it was used as intended.

## Introduction

Asthma is estimated to affect 358 million people worldwide^1^. In the UK, 8 million people have been diagnosed with asthma^2^. The goal of asthma management is optimal control of asthma symptoms, to reduce the risk of exacerbations, and for individuals to be able to lead a full and productive life^3,4^; however, this is not always achieved^5,6^. Despite the availability of effective treatments, asthma outcomes remain sub-optimal, resulting in many avoidable deaths, hospital admissions, quality of life impairment, and societal costs^5,7–9^. Clinical guidelines recommend promoting self-management through the provision of a personalised asthma action plan, attendance at annual asthma reviews, and correct inhaler technique use^10^. However, patient adherence to regular preventer medication, such as inhaled corticosteroids, is often low^11^, patients’ inhaler technique can be poor^12^, personal asthma action plans are underused, and annual asthma reviews are underattended^6,8^.

One potentially cost-effective method for promoting self-management is through digital interventions, which offer convenient 24-h access to relevant and personalised self-management support. There is preliminary evidence that digital interventions for asthma self-management can lead to improvements in asthma control and quality of life, with no evidence of harm^13,14^. However, there is currently a lack of robust evaluations of digital interventions for adults with asthma.

My Breathing Matters is an internet-based self-management intervention for asthma, which aims to improve the quality of life of adults with asthma through improved pharmacological (e.g., supporting medication adherence) and non-pharmacological (e.g., breathing retraining, stress reduction) self-management^15^. Other digital interventions for asthma focus on controlling asthma through pharmacological management or self-monitoring of physiological and behavioural data^14,16^. Unique to this intervention was the integration of non-pharmacological self-management, including breathing retraining^17^ and several previously evaluated interventions promoting healthy lifestyle behaviours (smoking cessation^18^, physical activity^19^, weight management^20^, and handwashing to prevent infections^21^). It was developed using theory-based, evidence-based, and person-based approaches to intervention development to maximise its effectiveness, feasibility and acceptability^15,22,23^. Trial feasibility outcomes (such as recruitment, retention, and randomisation) in our randomised controlled feasibility trial (RCT) with 88 adults with asthma showed that a definitive RCT is feasible^24^. In addition, we observed consistent trends with improvements in asthma-related patient-reported outcome measures, including quality of life and asthma control. Before a definitive trial can be carried out, it is important to ensure that the intervention is acceptable to its target group and used as intended, to maximise its effectiveness^25^.

To achieve this, we carried out a mixed methods process evaluation of My Breathing Matters embedded within the feasibility trial. Process evaluations can help support and refine an intervention’s ‘programme theory’, which describes how an intervention is expected to lead to its effects (mechanisms of impact), the key intervention components, and how these interact with contextual factors (e.g., population, setting)^26,27^. Users’ ‘engagement’ with digital interventions has been hypothesised to moderate the intervention’s influence on its mechanisms of impact^28^. Engagement has been defined in terms of the extent to which an intervention is used (e.g., amount, frequency), the user’s subjective experience of the intervention, and engagement with wider behavioural goals, such as behaviour change and self-management^28,29^. Engagement is influenced by the digital intervention itself (content and delivery) and the context in which the intervention is used^28^. In asthma, there is a lack of research on potential factors influencing engagement with digital interventions^13^.

We aimed to explore user engagement with My Breathing Matters by examining how participants in the feasibility study used the intervention, and exploring participants’ experiences of the intervention. To refine our programme theory, we sought to identify aspects of the intervention’s delivery and content, and contextual factors (any external factors that might interact with the intervention to produce variations in the outcome) that may strengthen or impede users’ engagement with the intervention.

## Results

### Participants

Intervention usage data were available for all 44 participants. The My Breathing Matters Satisfaction Questionnaire was administered to all 36 participants in the intervention group, who registered with the intervention (eight participants were given a hyperlink to the intervention, but did not register). Seventeen intervention users and one non-user (*n* = 18; 41%) agreed to be interviewed. Participants who did not take part either withdrew before their interview was due (*n* = 4; 9%), could not be contacted by phone or email after multiple attempts (*n* = 18; 41%) or were too busy (*n* = 4; 9%). Table 1 provides the participants’ demographics.

**Table 1:** Participant demographics at baseline.

	All intervention participants (*n* = 44)	Users (*n* = 36)	Non-users (*n* = 8)	Interviewed (*n* = 18)	Not-interviewed (*n* = 26)	
*Age*						
Mean (SD)	57.0 (14.2)	56.8 (15.1)	57.9 (10.3)	60.3 (13.2)	54.7 (14.7)	
Range	20–78	20–78	43–77	29–77	20–78	
*Gender n (%)*						
Female	27 (61.4)	23 (63.9)	4 (50.0)	12 (66.7)	15 (57.7)	
Male	17 (38.6)	13 (36.1)	4 (50.0)	6 (33.3)	11 (42.3)	
*Ethnicity n (%)*						
White	42 (95.5)	36 (100.0)	6 (75.0)	18 (100.0)	24 (92.3)	
Other	2 (4.5)	0	2 (25.0)	0	2 (7.7)	
*Baseline asthma quality of life (AQLQ) score*						
Mean (SD)	4.9 (0.9)	5.0 (0.9)	4.2 (0.8)	5.0 (1.1)	4.7 (0.8)	
*Time since asthma diagnosis (years)*						
Mean (SD)	25.2 (17.2)^a^	25.8 (18.0)^b^	22.8 (14.4)	24.6 (19.7)^c^	25.6 (15.8)	
Range	1.3–64.0^a^	1.3–64.0^b^	3.0–43.0	1.3–64.0^c^	3.0–64.0	
*Marital status (%)*						
Married	28 (63.6)	24 (66.7)	4 (50.0)	13 (72.2)	15 (57.7)	
Living with a partner	4 (9.1)	3 (8.3)	1 (12.5)	2 (11.1)	2 (7.7)	
Widowed	4 (9.1)	3 (8.3)	1 (12.5)	3 (16.7)	1 (3.8)	
Divorced	1 (2.3)	1 (2.8)	0	0	1 (3.8)	
Separated	2 (4.5)	1 (2.8)	1 (12.5)	0	2 (7.7)	
Single	5 (11.4)	4 (11.1)	1 (12.5)	0	5 (19.2)	
*Highest level of education (%)*						
Postgraduate qualification (e.g., Masters, PhD)	4 (9.1)	3 (8.3)	1 (12.5)	3 (16.7)	1 (3.8)	
Undergraduate qualification (e.g., Degree, HNC, and HND)	20 (45.5)	19 (52.8)	1 (12.5)	6 (33.3)	14 (53.8)	
Further education (e.g., A-Levels, ONC, and OND)	5 (11.4)	5 (13.9)	0	2 (11.1)	3 (11.5)	
School leaver (e.g., GCSEs, O-levels)	10 (22.7)	7 (19.4)	3 (37.5)	5 (27.8)	5 (19.2)	
No formal educational qualifications	5 (11.4)	2 (5.6)	3 (37.5)	2 (11.1)	3 (11.5)	
*Age left full-time education*						
Mean (SD)	19.4 (7.0)^d^	19.5 (6.8)^e^	18.9 (8.3)	20.7 (9.0)	18.4 (5.0)^f^	
*Internet use per week (hours)*						
Mean (SD)	13.1 (12.4)	13.2 (11.9)	12.9 (15.0)	12.4 (13.5)	13.7 (11.7)	

*GCSEs* general certificate of secondary education, *O-level* the general certificate of education ordinary level, *A-level* the general certificate of education advanced level, *ONC* ordinary national certificate, *OND* ordinary national diploma.

^a^*n* = 43.

^b^*n* = 35.

^c^*n* = 17.

^d^*n* = 42.

^e^*n* = 34.

^f^*n* = 24.

### How did participants in the feasibility trial use the intervention?

Of the intervention participants, 81.8% (*n* = 36) logged into My Breathing Matters at least once and between 1 and 25 times (Median = 4; IQR = 8). Those using the intervention more than once (*n* = 27) used it between 1.89 to 337.85 days (Median = 120.96; IQR = 148.23). Each session (total sessions = 231) lasted between 0.01 and 58.81 min (Median = 4.69; IQR = 8.33). Of the 34 participants who reached the core intervention content, most (73.5%; *n* = 25) looked at both the pharmacological and non-pharmacological content and most (71%; *n* = 24) chose to look at the non-pharmacological content first. Table 2 provides information on number of participants using each intervention component. The breathing retraining module was the most viewed component and over half of participants signed-up to the breathing retraining challenge tool. The other intervention tools were used by less than a third of participants.

**Table 2:** Numbers (and percentages) of participants who used each intervention component (*n* = 36).

Intervention component	Participants who viewed at least one page of the session *n* (%)	Participants who used the main tool in the session *n* (%)
*Pharmacological content*		
Medication advice	21 (58.3%)	n/a
4-week challenge	19 (52.8%)	10 (27.8%)^a^
Personal asthma action plan	16 (44.4%)	6 (16.7%)^b^
Annual asthma review	16 (44.4%)	1 (2.8%)^c^
*Non-pharmacological content*		
Breathing retraining	27 (79.4%)	20 (55.6%)^d^
Stress management	13 (36.1%)	6 (16.7%)^e^
Friends and family support	10 (27.8%)	1 (2.8%)^f^
*Lifestyle changes*		
Weight management	3 (8.3%)	n/a
Physical activity	3 (8.3%)	n/a
Handwashing	2 (7.7%)	n/a
Smoking cessation	0	n/a

^a^Signing up to the 4-week challenge.

^b^Viewed blank plan or made online plan.

^c^Booked an appointment with GP and recorded the appointment online.

^d^Signed-up to breathing retraining.

^e^Used the stress management tools.

^f^Emailed someone a link to the Friends and Family module.

### What were intervention participants’ experiences of the intervention?

**Fig. 1:**
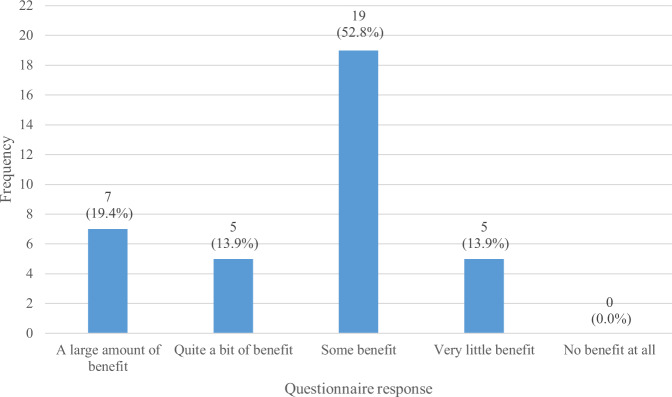
Benefits of My Breathing Matters. Participant responses to single item on My Breathing Matters Satisfaction Questionnaire relating to benefits of using My Breathing Matters.

**Fig. 2:**
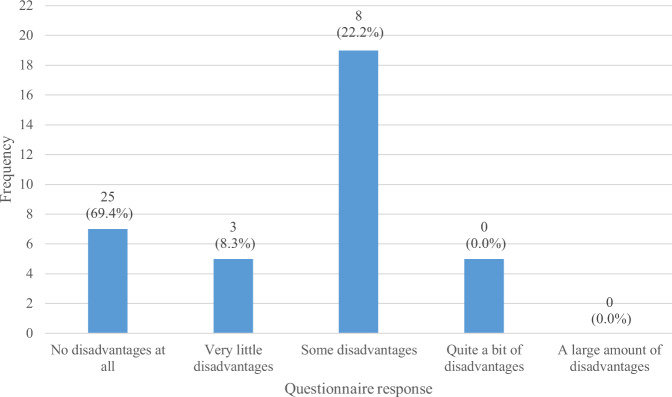
Disadvantages of My Breathing Matters. Participant responses to single item on My Breathing Matters Satisfaction Questionnaire relating to disadvantages of using My Breathing Matters.

**Fig. 3:**
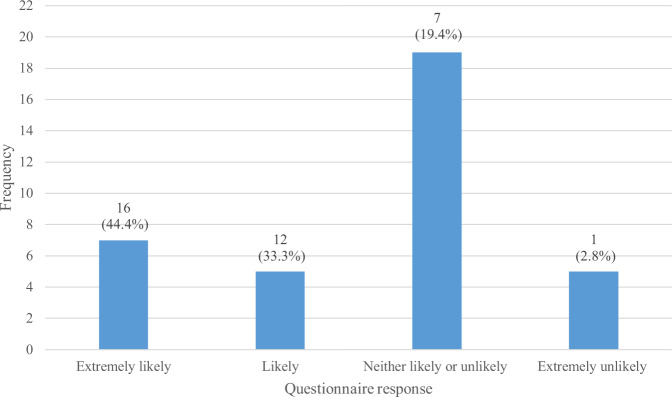
NHS Friends and Family Test. Participant responses to NHS friends and family test relating to how likely they would be to recommend My Breathing Matters to friends and family if they needed similar care and treatment.

In the My Breathing Matters Satisfaction Questionnaire, 86.1% (*n* = 31) of the intervention users (*n* = 36) reported that My Breathing Matters provided at least some benefit (Fig. 1) and 69.4% (*n* = 25) reported that there were ‘no disadvantages at all’ (Fig. 2). A large majority of survey respondents (77.8%; *n* = 28) reported that they would recommend My Breathing Matters to friends and family if they needed similar care and treatment (Fig. 3).

Content analysis of the free-text comments identified 14 benefits of using My Breathing Matters (*n* = 28; Table 3) and nine disadvantages (*n* = 13; Table 4). Information provision (*n* = 12) and provision of breathing retraining (*n* = 5) were the most commonly cited benefits. A dislike of the intervention’s design (*n* = 3) and that the intervention was not accessible on smartphones and computer tablets (*n* = 3) were the most commonly cited disadvantages.

**Table 3:** Content analysis of free-text comments regarding benefits of using My Breathing Matters (*n* = 28).

Code	Description	Frequency
Information provision	Intervention had improved awareness, improved or validated understanding about asthma and its management. Participants liked the lifestyle advice and tips on management. The information in the intervention was reliable and clear.	12
Provision of breathing retraining	The breathing exercises were cited as a benefit of using the intervention. The intervention helped them realise the benefits of the breathing exercises/correct breathing, learn correct breathing and be more aware of their breathing.	5
Medication adherence	The intervention helped people to build a medication habit, ‘take notice’ of medication, and made them realise they should be using a preventer inhaler regularly.	3
Lifestyle changes	The intervention provided lifestyle advice, including healthy eating, weight management, and physical activity. Participants had lost weight and increased their physical activity since using the intervention.	3
Reassurance	The intervention reassured people that their asthma symptoms were normal, that they were doing the right things to manage their asthma, and confirmed what they already knew.	3
Relaxation	The intervention helped people to relax. Participants started doing the relaxation techniques and they helped one participant get to sleep.	3
Access to information	The intervention provides access to information quickly and easily. The intervention can be accessed at home.	2
Control of asthma symptoms	The intervention helped people to control their asthma symptoms or improved their lung function.	2
Motivation for asthma self-management	The intervention makes people think more about their asthma and gives them to motivation to manage their asthma.	2
Provision of action plan	Being given access to an action plan/made aware of it.	2
Speaking to friends and family	Two participants had discussed asthma and its management with family and friends.	2
Dealing with triggers	The intervention helped one person deal with asthma triggers.	1
General health	The intervention made one person think about their general health, as well as asthma.	1
Support	The intervention made one participant feel they were being taken care of.	1

**Table 4:** Content analysis of free-text comments regarding disadvantages of using My Breathing Matters (*n* = 13).

Code	Description	Frequency
Disliked design	Aspects of the design participants disliked, in particular the unlocking feature.	3
Not accessible on their device	The intervention could not be accessed on phone or computer tablet.	3
Difficulties logging on	Participants experienced difficulties logging on.	2
Too many or too little emails	Participants received too many or not enough emails to keep engaged.	2
Annoying	The intervention was annoying or slow.	1
Boring	The intervention was short and became boring after a few months.	1
Lack of human contact	The intervention did not provide one-to-one human contact to allow the participant’s questions to be answered.	1
Patronising	The intervention was patronising.	1
Time consuming	Participant did not have time to do the breathing exercises during the day.	1

Thematic analysis of the qualitative interviews identified four themes, which are outlined alongside the codes in Supplementary Table 1.

The first theme was ‘Benefits of My Breathing Matters’. Many participants reported how they noticed changes in their asthma symptoms since using My Breathing Matters, including reduced coughing, chest tightness, and breathlessness; improved peak flow; feeling more in control of their asthma; and fewer or no asthma attacks.

I’m not coughing when I wake up in the morning any more, or rarely. I’m not waking up in the night feeling tight-chested and that I can’t breathe properly. (P14, 31–40 years old, female, asthma 21–30 years)

This change was mainly attributed to the breathing retraining and improved medication use. In contrast, some interview participants said that they did not notice any changes to their asthma since using the intervention.

Participants who used the 4-week medication challenge (see Table 5) explained how this component had helped them get into the habit of using their preventer inhaler and use their inhalers correctly.

**Table 5:** Description of My Breathing Matters intervention components.

Target behaviour	Description
Improved preventer medication adherence	• Information about the benefits of medication use for prevention of asthma symptoms. • Addressing ‘common concerns’ about asthma medication. • A 4-week challenge (in which users were encouraged to engage in habitual optimal preventer inhaler use) to help people develop positive medication habits.
Appropriate healthcare service use	• Tools to create and store a Personal Asthma Action Plan and provide encouragement for its use. • Provide encouragement to attend an annual Asthma Review.
Engagement with breathing retraining	• A breathing retraining programme^17^ to help control asthma symptoms, including videos on how to improve your breathing technique.
Engagement with stress management	• Provision of stress management techniques, including relaxation, and advice on stress management (e.g., time management) and adaptive ways of thinking (e.g., thought awareness, using positive thinking, talking through your worries), to reduce asthma-related stress.
Send information to friends and family to encourage them to engage in asthma management	• Ability to send friends and family a hyperlink to relevant information about asthma treatment and symptoms.
Lifestyle changes	• Access to previously developed lifestyle change programmes adapted for asthma, including: • StopAdvisor^18^ to support smoking cessation, • Getting Active^19^ to increase physical activity adapted for asthma, • POWeR^20^ to support weight management, • Germ Defence^21^ to promote handwashing to prevent infections.

I haven’t been terribly good at using the brown [preventer] inhaler. But I have pretty much got into the habit now and I would put that very much down to the website reminders. (P4, 61–70 years old, male, asthma 21–30 years).

Others reported how, since using My Breathing Matters, they had not needed to use their reliever inhaler as often. This was because they had not had any exacerbations, were using their preventer inhaler as prescribed, or started to practice the breathing techniques provided in the intervention when they were having symptoms instead.

Sometimes, I forget, you know, and I think, ‘Oh actually, perhaps I should have taken it [reliever inhaler]’, but then I think let’s do my breathing techniques. Sometimes I haven’t needed to take it…the website’s been good for that. (P1, 41–50 years old, female, asthma 21–30 years)

One participant reported how the intervention reassured them that it was acceptable to use their reliever when they need to (rather than just tolerating symptoms), while another had been told by a health professional that their asthma had improved to a point that meant they no longer needed to use their preventer inhaler.

Many participants spoke about how My Breathing Matters improved their breathing awareness, technique and posture.

I just feel that, sort of, before I used to breathe a lot through my mouth… And I find that, obviously, that now I’m breathing through my nose, my asthma’s not as bad… I find that I’m not coughing as much. (P12, 21–30 years old, female, asthma < 5 years)

Interview participants reported how My Breathing Matters had helped them to better identify, and deal with, asthma triggers (e.g., air pollution); gave them breathing and relaxation techniques to manage chest tightness and breathlessness; and prompted them to engage in healthy lifestyle changes (e.g., physical activity, healthy eating). A few participants explained how the intervention could help them to decide whether to seek health professional advice, and help them avoid unnecessary GP visits or burdening their healthcare team.

A few participants mentioned that their understanding of asthma and its treatment had improved. One participant learned how she should have had an asthma action plan, which she had printed and intended to take to her to asthma clinic.

It might have been useful if I’d had one of these [an action plan] years ago. Then I might have known what to do at the time [I had an asthma attack]. So that was extremely useful. (P8, 61–70 years old, female, asthma 5–10 years).

The action plan also prompted another participant to have conversations with their family about what they should do if he had an exacerbation and could not explain this to them at the time. On the other hand, some participants commented how they already knew a lot of the information, felt there was nothing new in the intervention, found some of the content repetitive, or believed the advice was common sense.

Some participants explained how the breathing retraining and stress management techniques helped them relax or stay calm, in particular when they were feeling tight chested, panicking when having an asthma attack, and for trying to get to sleep. A few participants explained how My Breathing Matters could make people think more positively about asthma, especially if you have just been diagnosed.

I think maybe that’s what I’ve really gained from it [My Breathing Matters], I’ve thought about it [asthma] more and if you think about problems or if you think about different things then that’s a good thing to, you know, you’re actively trying to improve something about it and, yeah, so I’m definitely thinking more positively. (P11, 41–50 years old, female, asthma 21–30 years)

Other benefits included addressing any asthma concerns you might have (e.g., side effects of medication, symptoms); providing reassurance that there are things that can help them cope; and highlighting that people with asthma are not alone and that there are other people with asthma or similar problems.

The second theme was ‘Views on the intervention content’. Participants particularly valued the breathing retraining, with many finding this the most helpful component. Most participants liked the videos and found the techniques relatively easy to learn. A few people found some of the techniques difficult to learn, including slow breathing and controlled breath holding, with one person preferring to have received the training in person. Another participant did not understand why the breathing exercises were beneficial and found the video irritating.

Some participants did not want to rely solely on their asthma medication to manage their asthma and liked that My Breathing Matters provided alternative management strategies, mainly the breathing retraining.

Anything that helps you only take the amount of medication you really need and helps you to self-control asthma in some way. And if My Breathing helps you to do that, that’s got to be a good thing. (P2, 61–70 years old, female, asthma 11–20 years)

Now, after using the website, it’s made me think about, well, what other things can I do to help myself, so that I don’t have to rely on my inhaler so much? (P12, 21–30 years old, female, asthma <5 years)

In the 4-week medication challenge (see Table 5), participants valued the email reminders, the advice about keeping their inhalers somewhere accessible as a reminder, and the realisation that it was benefiting them. The other intervention components (action plan, annual asthma review, stress management, and friends and family) were used to a lesser extent. Most participants either had not yet used the component or found that these components were not relevant to them. None of the interview participants reported contacting the Asthma UK helpline when asked about this.

The third theme was ‘Views on the intervention design’. Participants expressed positive views on the intervention design and found the content easy to understand. Some participants liked that it was designed by an experienced team and that it was associated with a national charity (Asthma UK), and felt that the information was authentic and high quality. Generally, people found the intervention easy to navigate. However, a few people experienced navigation and technical difficulties, including logging on, following URLs in emails, and accessing the intervention by phone or tablet, or on their workplace computer. Participants expressed mixed views regarding the unlocking feature of My Breathing Matters, whereby new content was made available to users over time. Some liked this feature as it structured their learning and stopped them from feeling overwhelmed by too much information, but others found it frustrating or did not understand the reasoning behind the feature.

I’d have been bombarded with it all if it was too much at once, so it was quite nice it came in sections slowly… it’s too much to take in otherwise. (P1, 41–50 years old, female, asthma 31–40 years)

I wanted to look through other bits that weren’t enabled and then had to wait for them… I think that probably would discourage me from using it. (P4, 61–70 years old, male, asthma 21–30 years)

Participants liked receiving regular emails with additional behavioural content from My Breathing Matters because they reminded them to take their medication and use the website, provided encouragement and additional advice, and facilitated quick access back to the website. A few people expressed negative views of the emails, including finding the email content irritating or not useful, or that it made them feel guilty for not using the website.

The forth theme was ‘Contextual factors influencing intervention engagement’. Participants’ engagement with My Breathing Matters was influenced by their perceptions of their asthma control. Participants explained how they did not engage with the intervention or specific components (e.g., the Asthma UK helpline, action plan, or the medication section) because they did not think their asthma was severe enough.

I possibly briefly looked at the sort of action plan thing, but decided that, actually, I didn’t think it was gonna be beneficial for me… I just thought that probably my asthma wasn’t severe enough that it was something that I needed to do at that moment in time. (P12, 21–30 years old, female, asthma <5 years)

Likewise, participants explained that they were more likely to use the intervention when their asthma symptoms were bad (e.g., in the winter or during allergy season), and less likely to use it when their asthma was well controlled. A few participants explained how, most of the time, they simply ‘forgot’ or tried not to ‘dwell’ on their asthma unless it was significantly restricting their lives.

My asthma is fairly well controlled, I haven’t needed to refer to the [My Breathing Matters] site… I’m very much a kind of person that, actually, I don’t dwell on, you know, things that might inhibit you in life, and just get on with life… You know, I’ve had far more worse than asthma. (P15, 61–70 years old, female, asthma 31–40 years)

On the other hand, two people were unlikely to use it if it their asthma was bad, instead choosing to seek medical attention.

Some participants explained that they did not use certain components because they did not consider them relevant. For example, they already practiced the recommended behaviours (e.g., taking medication, attending reviews, being more active), were not stressed (relating to the stress management techniques), or their family or friends already knew about asthma.

Participants explained how they thought My Breathing Matters would be most useful at the beginning of their asthma journey, once you have been diagnosed with asthma. Likewise, some people who have had asthma for a long time reported it was less useful. A few participants explained how they were not confident with using computers or expressed a dislike towards them. The non-user we interviewed was keen to use the intervention, but felt he lacked the computer skills to log onto it. Other reported reasons for low usage or not using certain components included lack of time or being busy with other priorities (e.g., work, family); and comorbidities that made some of the intervention behaviours (physical activity, breathing exercises) challenging.

### What factors may influence user engagement with My Breathing Matters?

**Fig. 4:**
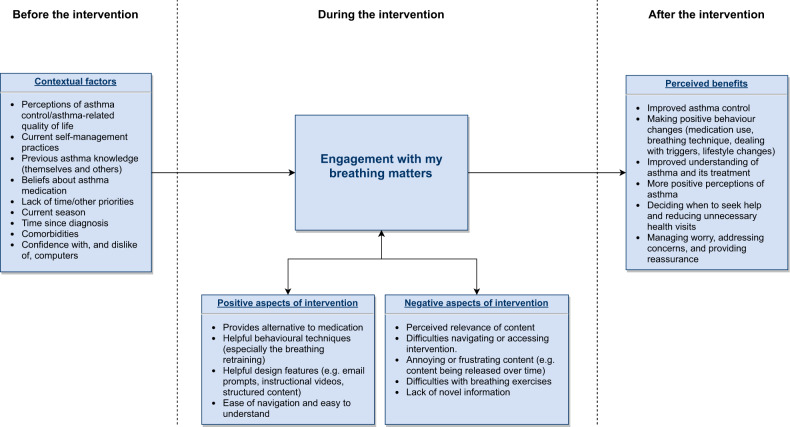
Summary of the qualitative findings. Demonstrating the contextual factors and aspects of the intervention content and delivery that may influence engagement with My Breathing Matters and the perceived benefits.

Across the qualitative findings, we identified several contextual factors and aspects of the intervention’s content and delivery that may influence user engagement with the intervention (Fig. 4). Contextual factors were derived from the interview data (theme 4) and included pre-existing beliefs (e.g., perceptions of asthma control/asthma-related quality of life, beliefs about medication), knowledge of asthma management and skills (e.g., confidence with computers), current self-management practices, environmental factors (current season, lack of time), and health status (time since diagnosis, comorbidities). The interview data and qualitative data from the My Breathing Matters Satisfaction Questionnaire highlighted aspects of the intervention’s content and delivery, that may influence engagement including appeal and perceived ease of the target behaviours (e.g., breathing retraining); appeal of the behaviour change techniques (e.g., email reminders) and design (e.g., content released over time, instructional videos); novelty, relevance and clarity of the intervention content; and ease of use (navigation and accessing the website). Users reported both positive and negative aspects, and both are summarised along with the perceived benefits of the intervention (derived from theme 1 of the interview data and the qualitative questionnaire data) in Fig. 4.

## Discussion

This mixed methods process evaluation study explored users’ engagement with My Breathing Matters, an internet-based self-management intervention for asthma. Overall, engagement with the intervention was high, it was used as intended, and people with asthma expressed positive views of the intervention, its components, and its design features; thus, demonstrating that it was acceptable to participants. Users reported experiencing several benefits of the intervention, mainly improved asthma control, medication use, and breathing technique. These perceived benefits were in line with the hypothesised intervention mechanisms of impact and outcomes outlined in our original logic model. Our study findings also extended our current programme theory by identifying aspects of the intervention (content and delivery), and contextual factors that may influence user engagement with the intervention.

Despite our attempts to engage those who did not perceive themselves as having active asthma and only recruiting those with impaired asthma-related quality of life, users still questioned the relevance of the intervention and its components, and did not believe that their asthma was severe enough for the intervention. This mirrors other studies that have demonstrated disparities between perceived and objective measures of asthma control, with patients overestimating how well their asthma was controlled^30,31^. Notably, user engagement in this study was high despite such beliefs. This may be due to our use of ‘positive illness contexts’ as a key intervention design feature (promoting health rather than preventing illness). In this way, even when users considered the intervention not specifically necessary for asthma control, My Breathing Matters still provided self-management support. Users reported several benefits of the intervention, and our feasibility study observed trends with improvement across a range of asthma outcomes^24^. This demonstrates that interventions developed using theory-based, evidence-based, and person-based approaches that target likely barriers to behaviour change can lead to effective user engagement and positive outcomes among individuals with different health beliefs, such as those in heterogeneous chronic disease populations.

Uniquely, My Breathing Matters integrated breathing retraining alongside established pharmacological self-management approaches. Consistent with other qualitative evaluations of breathing retraining^32,33^, users valued how the non-pharmacological approaches in My Breathing Matters could help reduce their reliance on medication, which is an important goal for people with asthma^34^. Most participants were satisfied with the online delivery of breathing retraining, with just a few users finding the exercises difficult to learn and only one participant reporting that they would have preferred to receive their training face-to-face with a health professional; thus further demonstrating the feasibility of delivering breathing retraining via an unguided digital intervention. A trial of breathing retraining demonstrated that face-to-face delivery was no more effective than DVD delivery^17^.

In an attempt to maximise user engagement and ensure all core content was accessed^31^, we implemented a design feature whereby new content was made available to users over time. Although this feature had been used successfully in other interventions^35^ and some study participants found this feature helpful, others found this feature frustrating and did not understand the rationale behind it. It may be that by restricting users’ access to specific content, the intervention may have impaired their sense of control and autonomy, which are important factors for maximising engagement^28^. In future versions of the intervention, it would be helpful to provide users with a strong rationale for this feature (e.g., to encourage people to practice the techniques they have already accessed before trying new techniques), but allow users to unlock additional content themselves if they wished to maximise user autonomy^22,28^ and avoid disengaging active users.

One strength of this study was its mixed methods design. The triangulation of questionnaire measures with qualitative interviews, and usage data enabled us to explore different aspects of intervention engagement and to increase the credibility of the research. Even though some questionnaires such as the My Breathing Matters Satisfaction Questionnaire were not formally validated, we could examine the extent to which the intervention is used, and users’ subjective experiences of using the intervention and enacting its target behaviours (e.g., breathing retraining). Due to the limited sample size of the feasibility trial (*n* = 88), we were not powered to do a more in-depth analysis of the usage data. A fully powered RCT is needed to explore how process measures, such as perceptions of asthma, pre-intervention levels of medication adherence, and time since diagnosis, is associated with user engagement and asthma outcomes. It would also be worthwhile exploring how usage might change across the seasons, given that some participants explained how they were more likely to use the intervention during certain seasons, when their asthma symptoms were worse. Although we endeavoured to recruit participants across a broad demographic range, participants were generally older and white, and had high levels of educational attainment. They were also recruited from a feasibly trial sample, so are unlikely to be representative of the wider asthma population^36^. A wider reach would avoid further worsening the digital divide and health inequalities. Moreover, the small sample size of the feasibility study meant that we could not purposively sample participants based on their usage and were, therefore, only able to recruit one non-user. A larger sample size would have allowed us to better target and capture the views of non-users, and those who were less engaged with the intervention. Interviews with the control group would have allowed us to explore their experiences with usual care, in order to explore which perceived benefits are unique to the intervention and not from the feasibility trial itself. Interviews with those who declined to take part in the trial would have also given us useful insights into their reasons for this, and how user engagement might be improved^37^.

Our findings demonstrated that My Breathing Matters is acceptable and engaging to its target group, and the intervention was delivered and worked as intended. The person-based approach to intervention development was key to maximising intervention engagement and acceptability for adults with asthma. Along with the findings from the feasibility trial, the current study supports the move towards a fully powered RCT, including a mediation and moderation analysis, with only minor modifications to the intervention content required. More broadly, our findings highlight aspects of intervention content and delivery (such as targeting key issues using person-based approaches, providing non-pharmacological self-management approaches), and contextual factors (such as perceptions of asthma control, current self-management practices) that may influence user engagement with digital asthma interventions. These should be considered when implementing the intervention or when developing asthma behaviour change interventions.

## Methods

### Design

A convergent mixed methods design was used for the process evaluation in which qualitative and quantitative methods are implemented in the same research phase and given equal weight, but the data is analysed separately^38^. The process evaluation was embedded in a feasibility RCT of My Breathing Matters. Trial participants were randomised into an intervention group who were given access to My Breathing Matters or a usual care group. Outcome measures were assessed at baseline, 3 months and 12 months. Further details on the trial methods and feasibility outcomes are available elsewhere^24^. Quantitative usage data were collected to describe patterns of intervention usage over the 12-month study period. The My Breathing Matters Satisfaction Questionnaire was devised for this study and administered to intervention participants at 12-month follow-up to assess their satisfaction with the intervention. Qualitative interviews were carried out to explore intervention participants’ views and experiences of My Breathing Matters. Ethical approval was granted by the University of Southampton and South Central—Berkshire Research Ethics Committee (REC reference: 16/SC/0614). To increase the transferability of the research, the COREQ checklist^39^ was used to guide reporting of the qualitative research (Supplementary Table 2), and ensure a rich description of the participants and the research process.

### Intervention

My Breathing Matters was systematically developed using person-based, evidence-based, and theory-based approaches, drawing upon primary mixed methods research^17,31,40^, quantitative^14^ and qualitative^41^ systematic reviews, and consultation with Public and Patient Involvement (PPI) representatives and clinical and intervention development experts.

Following a person-based approach^22^, guiding principles were created, including intervention design objectives and design features to address key issues, needs, and behavioural challenges of the target population identified in the evidence synthesis stage. One key behavioural issue that emerged from the literature search conducted in the intervention development phase is that some people with non-optimal asthma control do not consider themselves as people with active asthma^42–44^. Therefore, one intervention design objective was to specifically engage this group. To do this, the intervention maintained a positive illness context throughout (referring to ‘keeping breathing healthy’ rather than ‘preventing asthma symptoms’), provided optional and flexible support only when needed, and promoted the belief that impaired quality of life can be improved (Supplementary Fig. 1). To target influences on asthma control that are not often acknowledged, such as anxiety, stress and lifestyle (e.g., smoking, obesity, and avoidance of physical activity), other design objectives aimed to encourage users to engage in non-pharmacological (e.g., breathing retraining, stress management, and lifestyle changes), as well as pharmacological self-management, to improve asthma control (see Yardley et al.^15^ for this process in more detail).

**Fig. 5:**
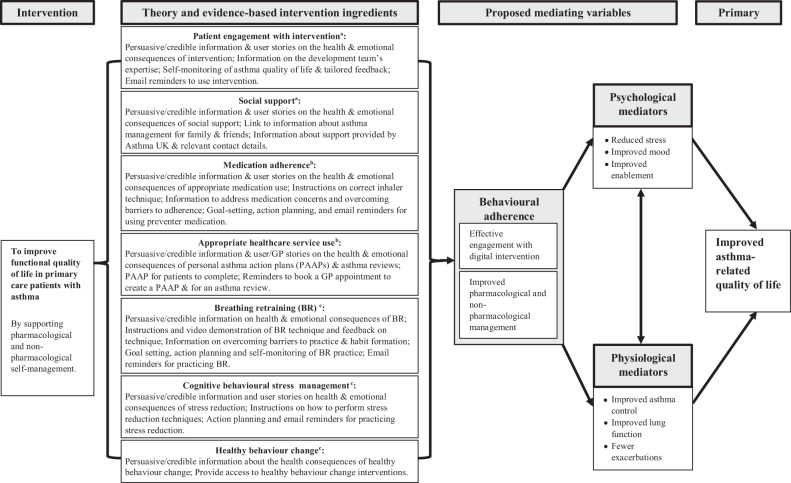
Logic Model of My Breathing Matters intervention. ^a^Uptake and engagement facilitation; ^b^Pharmacological support; ^c^Non-pharmacological support.

Theory-based behaviour analysis was used to identify the influences on target behaviours and the intervention components that could address these, and describe the intervention in terms of existing theory and programme level theory. A logic model was created to illustrate the hypothesised mechanisms of impact that explain how My Breathing Matters is expected to lead to improvements in asthma-related quality of life (Fig. 5). My Breathing Matters is hypothesised to improve asthma-related quality of life through behavioural adherence (improved pharmacological and non-pharmacological management, engagement with the intervention), improved physiological outcomes (asthma control, lung function, and exacerbations), and improved psychological outcomes (stress, mood, and enablement). Table 5 outlines the components of the intervention in more detail.

An intervention prototype was developed and, consistent with a person-based approach, the views and experiences of adults with asthma who used the intervention were explored using iterative qualitative methods (think aloud and retrospective semi-structured interviews), and the intervention was modified in response to this feedback.

On each unique login, users were asked to complete a brief quality of life assessment measuring activities, sleep, stress, illness, and reliever medication use (Supplementary Fig. 2). Based on their answers, users were signposted to relevant content. Content was not available all at once, rather different content was ‘unlocked’ at various time points after the user’s first visit to the website to encourage long-term engagement with the intervention (Supplementary Fig. 3). The intervention is self-directed, but the contact details for the Asthma UK helpline were given to provide additional support if required. The intervention is available at mybreathingmatters.co.uk.

### Participants and recruitment

Participants were eligible for the feasibility trial if they were aged 18 years or over, had physician-diagnosed asthma managed in primary care, had received at least one anti-asthma medication prescription in the previous year, and could use the Internet (self-judged). Anti-asthma medication included all commonly used inhaled and oral preparations for asthma treatment (both regular medication and as-required reliever preparations), such as inhaled corticosteroids, long and short acting beta agonists and leukotriene receptor antagonists. No patients were receiving injected biological treatments or maintenance oral corticosteroids. Participants also needed to have an impaired asthma-related health status at baseline, defined as a Mini Asthma Quality of Life Questionnaire^45^ score of less than 5.5. Full trial inclusion and exclusion criteria are described elsewhere^24^.

Eligible participants were identified and invited to take part in the trial by seven general practices from the Wessex, UK primary care research network. After the 3-month follow-up, all intervention group participants (*n* = 44) were approached by phone or email by a member of the study team and were invited to take part in a qualitative interview, irrespective of whether they used the intervention. Drawing on the guidelines on information power in qualitative interview studies^46^, we aimed to recruit approximately 20 participants to the interview study. This number was deemed adequate given the study’s narrow aim (views on one intervention), the small source population (*n* = 44), the specificity of the experiences, knowledge and properties among the intervention trial participants, and the likely high quality of dialogue from using an experienced qualitative researcher. Informed consent was obtained for all trial participants. Participants received a £10 shopping voucher for submitting their follow-up questionnaires at 12 months. Interview participants did not receive any additional incentives for taking part.

### Data collection

Intervention usage was automatically collected by the LifeGuide software (https://www.lifeguideonline.org), which was used to create and host the intervention. Data were collected on the number and duration of logins, date of last login, and pages visited. Participants were informed that they could use the intervention as much or as little as they liked.

The My Breathing Matters Satisfaction Questionnaire (Supplementary Note 1) was administered by paper to those who registered with the intervention at the 12-month follow-up appointment with a research nurse. Better understanding of the potential benefits and burdens of health interventions can help us to optimise these interventions and improve their effectiveness^47,48^. To explore these two aspects, we devised two items to assess benefits gained from using the intervention and disadvantages of the intervention, and open questions allowed participants to report any benefits and disadvantages. These items were developed in discussion with our multidisciplinary intervention development team, consisting of experts in intervention development and evaluation, behavioural science, and health economics. The one-item NHS Friends and Family Test^49^ assessed how likely participants are to recommend the intervention to friends and family, if they needed similar care and treatment using a 5-point Likert scale ranging from ‘extremely likely’ to ‘extremely unlikely’, with a ‘don’t know’ option. This tool is used by NHS England to assess patient satisfaction across a wide range of services.

For the qualitative interviews, a semi-structured interview schedule was developed by experts in health psychology (KG, BA, and LY) and asthma (MT, BA, and AB), and a PPI representative with asthma (DR). Interview questions were designed to explore the key functions for process evaluation outlined in the Medical Research Council process evaluation guidelines^50^: implementation (what was delivered), mechanisms of impact, and contextual factors. Specifically, the questions explored participants’ experiences of the intervention and its components, how they used the intervention, their perceived advantages and disadvantages of the intervention, times they were more and less likely to use the intervention, and reasons for any non-usage (See Supplementary Note 2 for interview schedule). Open-ended questions were used to ascertain the most important issues or challenges for participants.

Interviews were carried out by telephone by KG (female health psychologist and research fellow who was experienced in qualitative research) who was not involved in intervention development, and did not know the participants prior to the interviews. Participants were told that the interviews aimed to explore their view and experiences to help improve the research and intervention for future users. Interviews took place between July 2017 and January 2018, lasted between 21–65 min, were audio-recorded, and transcribed verbatim.

### Data analysis

The intervention usage data and the closed questions of the My Breathing Matters Satisfaction Questionnaire were analysed using descriptive statistics to describe patterns of intervention usage. Content analysis^51^ was carried out on the open question data to identify benefits and disadvantages of using the intervention.

The qualitative interviews were analysed using inductive thematic analysis^51,52^. Data analysis was assisted by QSR’s NVivo 11 qualitative data analysis software (QSR International Pty Ltd., 2017). Analysis was informed by guidelines for establishing trustworthiness in qualitative research^53–56^. KG familiarised herself with the data through repeated reading of the transcripts. Initial codes were generated that were grounded in the data and a coding manual was developed that listed all codes and themes, including descriptions and example quotes from the text. To increase the credibility of the research, the final coding manual was discussed and agreed with two other researchers (BA and LY) and the final interpretations in the results section were reviewed and agreed by all authors, as well as two PPI representatives. The constant comparison method^57^, a grounded theory technique, was used to compare codes across different participants, contexts, and situations. Disconfirming case analysis^54^ was used to actively identify data that did not fit with the identified themes. These two techniques were used to ensure the analysis was carried out with rigour and to increase its credibility. Participant quotes were used in the final write-up to illustrate the themes and pseudonyms used to refer to these participants. Data saturation was considered reached because participants in later interviews did not indicate any significant new benefits, concerns, or barriers to engagement with My Breathing Matters.

Once the qualitative analysis was complete, we reviewed key findings from the interviews and the My Breathing Matters Satisfaction Questionnaire to identify contextual factors, and aspects of the intervention’s content and delivery that may have influenced user engagement with the intervention.

### Reporting summary

Further information on research design is available in the Nature Research Reporting Summary linked to this article.

## Supplementary information

Supplementary Information

Reporting Summary

## Data Availability

The data that support the findings of this study are available from the corresponding author upon reasonable request.
