# Supplementary material for: Mixed methods process evaluation of my breathing matters, a digital intervention to support self-management of asthma
Source: NPJ Prim Care Respir Med. 2021 Jun 4;31:35. doi: 10.1038/s41533-021-00248-6 (PMC8178311; doi:10.1038/s41533-021-00248-6)
Supplement: Supplementary file 1 — Supplementary Information [file 41533_2021_248_MOESM1_ESM.pdf]

Supplementary Material

Supplementary Table 1 Themes and codes from data analysis for qualitative interviews

Theme	Codes
Benefits of My Breathing Matters	Improved asthma & symptoms
	No noticeable change in asthma or breathing
	Reduction in medication use
	Improved medication adherence
	Provides an alternative to medication
	Improved breathing technique & posture
	Identifying, and dealing with, asthma triggers
	Prompts lifestyle changes
	Managing breathlessness and chest tightness
	Facilitates self-management
	Improved knowledge of asthma and its treatment
	Better use of healthcare resources
	Relaxation
	Thinking more positively about asthma
	Provides reassurance
	Feeling less alone
Views on the intervention content	Views on 4-week challenge
	Views on personalised asthma action plan (PAAP)
	Views on asthma review
	Views on breathing retraining
	Views on stress management

Views on the intervention design	Views on Friends & Family section<br>Views on lifestyle modules<br>Views on Asthma UK & helpline<br>Views on website appearance<br>Views on intervention credibility<br>Views on delivery format<br>Views on emails<br>Views on information and advice<br>Views on information novelty<br>Views on interactive features<br>Views on usability<br>Views on information architecture
Contextual factors influencing intervention engagement	Relevance of intervention components<br>Perceptions of asthma severity<br>Time since diagnosis<br>Confidence with, and dislike of, computers<br>Season<br>Other priorities<br>Other health problems

Supplementary Table 2 COREQ checklist for qualitative interviews

No	Item	Guide questions/description	Comments	Location in manuscript
**Domain 1: Research team and reflexivity**				
Personal Characteristics				
1.	Interviewer/facilitator	Which author/s conducted the interview or focus group?	KG	Methods (Data collection)
2.	Credentials	What were the researcher's credentials? *E.g. PhD, MD*	KG – PhD, CPsychol.	Methods (Data collection)
3.	Occupation	What was their occupation at the time of the study?	KG – Health Psychologist & Research Fellow	Methods (Data collection)
4.	Gender	Was the researcher male or female?	Female	Methods (Data collection)
5.	Experience and training	What experience or training did the researcher have?	Experienced qualitative postdoctoral researcher.	Methods (Data collection)
Relationship with participants				
6.	Relationship established	Was a relationship established prior to study commencement?	Participants were not known to the researcher.	Methods (Data collection)

No	Item	Guide questions/description	Comments	Location in manuscript
7.	Participant knowledge of the interviewer	What did the participants know about the researcher? e.*g. personal goals, reasons for doing the research*	Participants were told that the interviews aimed to explore their view and experiences to help improve the research and intervention for future users.	Methods (Data collection)
8.	Interviewer characteristics	What characteristics were reported about the interviewer/facilitator? e.g. *Bias, assumptions, reasons and interests in the research topic*	The researcher was not involved in intervention development, although she was part of the same digital research team, which may have been a source of bias.	Methods (Data collection)
**Domain 2: study design**				
Theoretical framework				
9.	Methodological orientation and Theory	What methodological orientation was stated to underpin the study? *e.g. grounded theory, discourse analysis, ethnography, phenomenology, content analysis*	Thematic analysis.	Methods (Data collection)
Participant selection				

No	Item	Guide questions/description	Comments	Location in manuscript
10.	Sampling	How were participants selected? *e.g. purposive, convenience, consecutive, snowball*	All intervention participants from the feasibility trial were approached.	Methods (Participants and recruitment)
11.	Method of approach	How were participants approached? e.*g. face-to-face, telephone, mail, email*	All intervention group participants were approached by phone or email by a member of the study team and were invited to take part.	Methods (Participants and recruitment)
12.	Sample size	How many participants were in the study?	18	Results (Participants)
13.	Non-participation	How many people refused to participate or dropped out? Reasons?	Participants who did not take part either withdrew before their interview was due (*n*=4; 9%), could not be contacted by phone or email after multiple attempts (*n*=18; 41%) or were too busy (*n*=4; 9%).	Results (Participants)
Setting				
14.	Setting of data collection	Where was the data collected? e.*g. home, clinic, workplace*	Telephone	Methods (Data collection)

No	Item	Guide questions/description	Comments	Location in manuscript
15.	Presence of non-participants	Was anyone else present besides the participants and researchers?	No	-
16.	Description of sample	What are the important characteristics of the sample? *e.g. demographic data, date*	Demographic information can be found in Table 1. Interviews took place between July 2017 and January 2018	Table 1; Methods (data collection)
Data collection				
17.	Interview guide	Were questions, prompts, guides provided by the authors? Was it pilot tested?	The interview schedules can be found in Supplementary Note 2. It was reviewed a PPI representative.	Supplementary Note 2
18.	Repeat interviews	Were repeat interviews carried out? If yes, how many?	Only single interviews were carried out.	-
19.	Audio/visual recording	Did the research use audio or visual recording to collect the data?	Audio	Methods (Data collection)
20.	Field notes	Were field notes made during and/or after the interview or focus group?	Field notes were not made.	-

No	Item	Guide questions/description	Comments	Location in manuscript
21.	Duration	What was the duration of the interviews or focus group?	Between 21-65 minutes	Methods (Data collection)
22.	Data saturation	Was data saturation discussed?	Data saturation was considered reached because participants in later interviews did not indicate any significant new benefits, concerns or barriers to engagement with My Breathing Matters.	Methods (Data analysis)
23.	Transcripts returned	Were transcripts returned to participants for comment and/or correction?	No	-
**Domain 3: analysis and findings**				
Data analysis				
24.	Number of data coders	How many data coders coded the data?	One (KG) but the coding manual was discussed and agreed with two other researchers (BA & Y)	Methods (Data analysis)

No	Item	Guide questions/description	Comments	Location in manuscript
25.	Description of the coding tree	Did authors provide a description of the coding tree?	Yes in Supplementary Table 1	Supplementary Table 1
26.	Derivation of themes	Were themes identified in advance or derived from the data?	Derived from the data (inductive analysis).	Methods (Data analysis)
27.	Software	What software, if applicable, was used to manage the data?	QSR's NVivo 11 was used.	Methods (Data analysis)
28.	Participant checking	Did participants provide feedback on the findings?	No, but the final interpretations were reviewed and agreed with two PPI representatives.	Methods (Data analysis)
Reporting				
29.	Quotations presented	Were participant quotations presented to illustrate the themes / findings? Was each quotation identified? e.*g. participant number*	Participant quotations are presented and each quotation is identified by a pseudonym and their gender, age and asthma duration is noted.	Results (Qualitative interviews)
30.	Data and findings consistent	Was there consistency between the data presented and the findings?	Yes	Results (Qualitative interviews)

No	Item	Guide questions/description	Comments	Location in manuscript
31.	Clarity of major themes	Were major themes clearly presented in the findings?	Yes	Results (Qualitative interviews)
32.	Clarity of minor themes	Is there a description of diverse cases or discussion of minor themes?	Diverse cases are discussed.<br><br>Coding tree presented in in Supplementary Table 1.	Results (Qualitative interviews); Supplementary Table 1

Supplementary Table 3 SRQR checklist for qualitative interviews

No	Item	Description	Location in manuscript
**Title and abstract**			
1.	Title	Concise description of the nature and topic of the study. Identifying the study as qualitative or indicating the approach (e.g., ethnography, grounded theory) or data collection methods (e.g., interview, focus group) is recommended.	Title identifies research as a mixed methods study, which includes qualitative research.
2.	Abstract	Summary of key elements of the study using the abstract format of the intended publication; typically includes background, purpose, methods, results, and conclusions	Abstract formatted as per npj Primary Care Respiratory Medicine guidelines.
Introduction			
3.	Problem formulation	Description and significance of the problem/phenomenon studied; review of relevant theory and empirical work; problem statement.	Given in introduction.

No	Item	Description	Location in manuscript
4.	Purpose or research question	Purpose of the study and specific objectives or questions	Aims given in last paragraph of introduction.
Methods			
5.	Qualitative approach and research paradigm	Qualitative approach (e.g., ethnography, grounded theory, case study, phenomenology, narrative research) and guiding theory if appropriate; identifying the research paradigm (e.g., post-positivist, constructivist/interpretivist) is also recommended; rationale.	Mixed methods research; inductive thematic analysis. Approach detailed in the data analysis section.
6.	Researcher characteristics and reflexivity	Researchers' characteristics that may influence the research, including personal attributes, qualifications/experience, relationship with participants, assumptions, and/or presuppositions; potential or actual interaction between researchers' characteristics and the research questions,	Characteristics of the interviewer, including credentials, relationship with participants and involvement in intervention development, given in data collection section.

No	Item	Description	Location in manuscript
		approach, methods, results and/or transferability.	
7.	Context	Setting/site and salient contextual factors; rationale.	Feasibility trial participants recruited from primary care. Interviews carried out by telephone. Detail given in participants and recruitment section.
8.	Sampling strategy	How and why research participants, documents, or events were selected; criteria for deciding when no further sampling was necessary (e.g., sampling saturation); rationale.	Study was nested within a feasibility randomised controlled trial study. All participants from intervention arm were approached. Data saturation was considered reached because participants in later interviews did not indicate any significant new benefits, concerns or barriers to engagement with My Breathing Matters. Detail given in participants and recruitment section.
9.	Ethical issues pertaining to human subjects	Documentation of approval by an appropriate ethics review board and participant consent, or explanation for lack thereof; other	Ethical approval was granted and details are reported in the Design section.

No	Item	Description	Location in manuscript
		confidentiality and data security issues.	
10.	Data collection methods	Types of data collected; details of data collection procedures including (as appropriate) start and stop dates of data collection and analysis, iterative process, triangulation of sources/methods, and modification of procedures in response to evolving study findings; rationale.	All data collection methods, including details of the interview and start and stop dates, given in the data collection section.
11.	Data collection instruments and technologies	Description of instruments (e.g., interview guides, questionnaires) and devices (e.g., audio recorders) used for data collection; if/how the instrument(s) changed over the course of the study	The interview schedules can be found in Supplementary Note 2. Interviews were audio-recorded (details in data collection section).
12.	Units of study	Number and relevant characteristics of participants, documents, or	Demographic information can be found in Table 1.

No	Item	Description	Location in manuscript
		events included in the study; level of participation.	
13.	Data processing	Methods for processing data prior to and during analysis, including transcription, data entry, data management and security, verification of data integrity, data coding and anonymization / de-identification of excerpts	Transcription, use of pseudonyms, and data handling approach is outlined in the data collection and analysis sections.
14.	Data analysis	Process by which inferences, themes, etc. were identified and developed, including the researchers involved in data analysis; usually references a specific paradigm or approach; rationale.	Content and thematic analysis approaches are outlined in the data analysis sections. Structure of codes and themes provided in Supplementary Table 1.
15.	Techniques to enhance trustworthiness	Techniques to enhance trustworthiness and credibility of data analysis,(e.g., member checking, triangulation, audit trail); rationale	Techniques to enhance trustworthiness is outlined in the data analysis and strengths and limitations section.
Results/Findings			

No	Item	Description	Location in manuscript
16.	Synthesis and interpretation	Main findings (e.g., interpretations, inferences, and themes); might include development of a theory or model, or integration with prior research or theory	A diagram of the main qualitative findings is presented in Figure 4 and the findings are discussed in relation to prior research in the discussion.
17.	Links to empirical data	Evidence (e.g., quotes, field notes, text excerpts, photographs) to substantiate analytic findings.	Anonymised quotes are provided throughout the results section to support the qualitative themes.
Discussion			
18.	Integration with prior work, implications, transferability, and contribution(s) to the field	Short summary of main findings, explanation of how findings and conclusions connect to, support, elaborate on, or challenge conclusions of earlier scholarship; discussion of scope of application/generalizability; identification of unique contribution(s) to scholarship in a discipline	The discussion explains how the findings support and build on previous research and highlights the unique contribution of this research.

No	Item	Description	Location in manuscript
		or field.	
19.	Limitations:	Trustworthiness and limitations of findings	The strengths and limitations are outlined in the discussion. Further details of the steps taken to increase the trustworthiness of the research is outlined in the data analysis section.
Other			
20.	Conflicts of interest	Potential sources of influence or perceived influence on study conduct and conclusions; how these were managed.	Competing interests are declared at the end of the manuscript.
21.	Funding	Sources of funding and other support; role of funders in data collection, interpretation, and reporting	Sources of funding are detailed in the acknowledgments section.

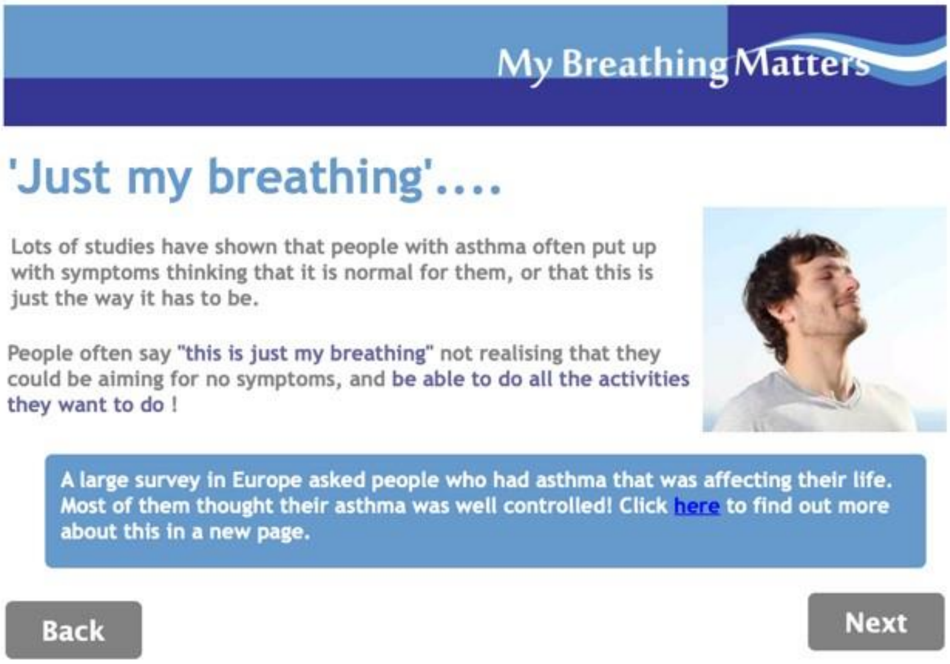

Supplementary Figure 1: Screenshot of My Breathing Matters pages to engage people who do not view themselves as having active asthma

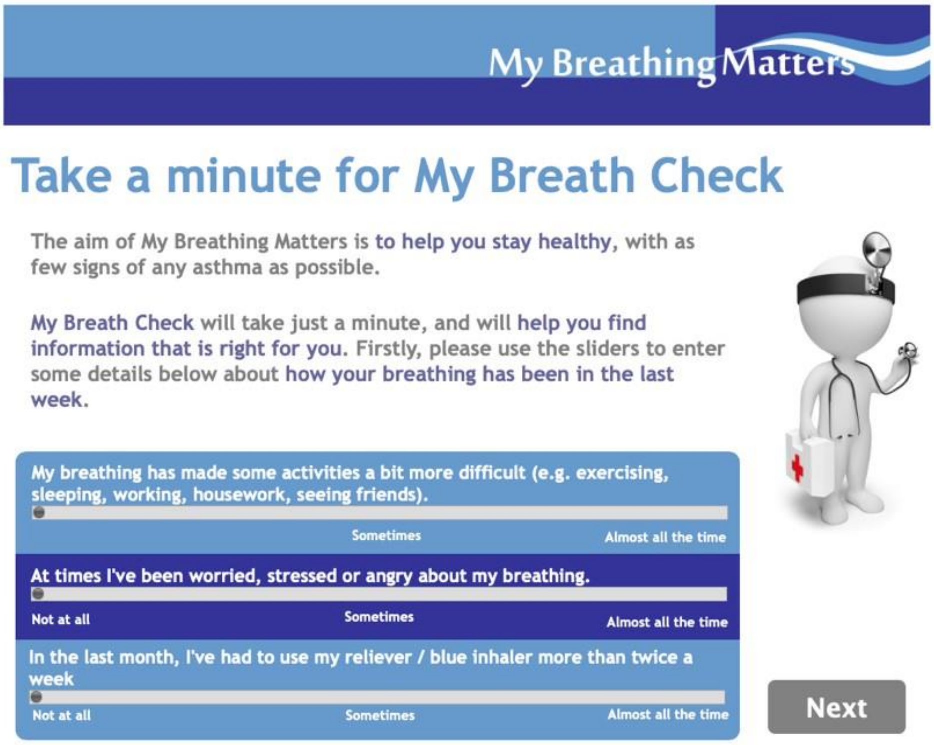

Supplementary Figure 2: Screenshot of the My Breathing Matters breath check

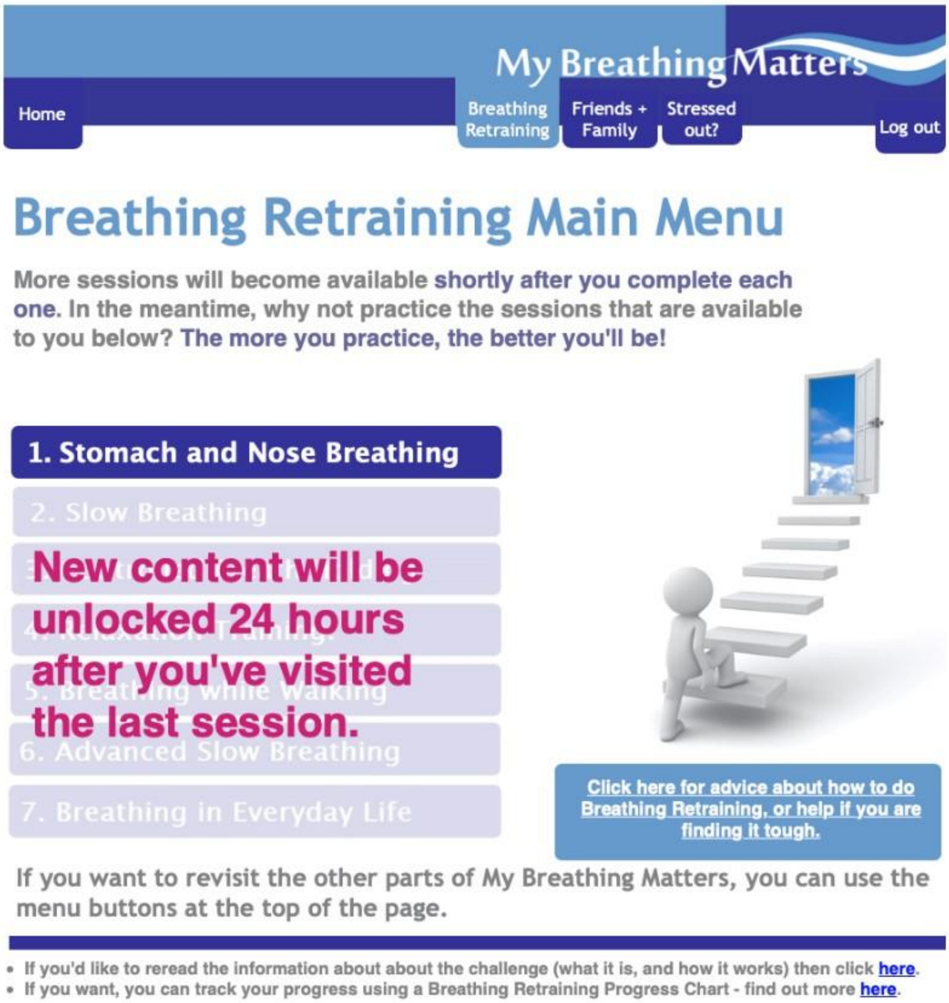

Supplementary Figure 3: Screenshot of My Breathing Matters breathing retraining with 'unlocking' feature

Supplementary Note 1: My Breathing Matters Satisfaction Questionnaire

INSTRUCTIONS: Please only answer the below questions **if you registered with the My Breathing Matters website in the last 12 months**. Please tick **one** answer for each question.

1. **Did you think there were any benefits of using My Breathing Matters?**

   No benefit at all ………………………….. ☐

   Very little benefit ………………………… ☐

   Some benefit ……………………………. ☐

   Quite a bit of benefit ……………………. ☐

   A large amount of benefit …………….. ☐

   If any benefits, please note them down below:

2. **Did you think there were any disadvantages of using My Breathing Matters?**

   No disadvantages at all ………………... ☐

   Very little disadvantages ……………... ☐

   Some disadvantages ……………………. ☐

   Quite a bit of disadvantages ………… ☐

   A large amount of disadvantages …. ☐

If any disadvantages, please note them down below:

3. How likely are you to recommend My Breathing Matters to friends and family if they needed similar care and treatment?

Extremely likely ………………………….. ☐

Likely ……………………………………. ☐

Neither likely or unlikely …………….. ☐

Extremely unlikely ……………………… ☐

Don't know ………………………………. ☐

Supplementary Note 2: Interview schedule

Interview questions for intervention participants who have logged on

Q1. Can you tell me what it's like to have asthma?

Q2. I'm really interested in hearing about your experiences of using My Breathing Matters, can you tell me all about it?

Q3. Can you tell me about anything you liked about My Breathing Matters?

Q4. Can you tell me about anything you disliked about My Breathing Matters?

Q5. Can you tell me about any advantages of using My Breathing Matters for you?

Q6. Can you tell me about any disadvantages of using My Breathing Matters for you?

Q7. The research will continue for another 9 months. Do you think you will keep on using My Breathing Matters over this time? *[Prompts: Why/why not?]*

Q8. Would you recommend My Breathing Matters to other people with asthma? *[Prompts: Why/Why not?]*

Q9. Since using My Breathing Matters, how do you feel about your asthma now?

Q10. Can you tell me about anything that you feel has changed from using My Breathing Matters?

a. Can you tell me about what changed? (e.g. anything different in your day-to-day life, the way you are managing your asthma?)

b. Can you tell me how you came to notice things changing?

c. Why do you think these things changed?

Q11. When do you think My Breathing Matters would be most helpful to you?

Q12. When do you think My Breathing Matters would not be helpful to you?

<u>For each component:</u>

Q13. **[If didn't use]** Can you tell me why you decided not to use this part of My Breathing Matters?

Q14. **[If used]** Can you tell me about how you found this section? *[Prompts: What did you like/dislike? Can you tell me about any problems you came across when doing the challenge?]*

<u>Emails:</u>

Q15. Can you tell me about how you felt when My Breathing Matters sent you emails?

Q16. Can you tell me what you thought about what the emails said?

Q17. Can you tell me your thoughts about how often you received the emails?

Q18. Can you tell me about any other ways you would like My Breathing Matters to contact you?

Q19. Can you tell me about any advantages of getting these emails?

Q20. Can you tell me about any disadvantages of getting these emails?

Interview questions for intervention participants who have not logged on

Q1. Can you tell me what it's like to have asthma?

Q2. We are interested to hear from people who did not use My Breathing Matters, can you tell me why you have not used My Breathing Matters?

Q3. What are your thoughts on using a website to help you to manage your asthma?

Q4. The research will continue for another 9 months. Do you think you will use My Breathing Matters over this time? *[Prompts: Why/why not?]*
