# Supplementary material for: Mixed methods process evaluation of my breathing matters, a digital intervention to support self-management of asthma
Source: NPJ Prim Care Respir Med. 2021 Jun 4;31:35. doi: 10.1038/s41533-021-00248-6 (PMC8178311; doi:10.1038/s41533-021-00248-6)
Supplement: Supplementary file 2 — Reporting Summary [file 41533_2021_248_MOESM2_ESM.pdf]

nature research

Corresponding author(s): NPJPCRM-01135R

Last updated by author(s): Feb 12, 2021

Reporting Summary

Nature Research wishes to improve the reproducibility of the work that we publish. This form provides structure for consistency and transparency in reporting. For further information on Nature Research policies, see our Editorial Policies and the Editorial Policy Checklist.

Statistics

For all statistical analyses, confirm that the following items are present in the figure legend, table legend, main text, or Methods section.

n/a	Confirmed	
☐	☒	The exact sample size (*n*) for each experimental group/condition, given as a discrete number and unit of measurement
☒	☐	A statement on whether measurements were taken from distinct samples or whether the same sample was measured repeatedly
☒	☐	The statistical test(s) used AND whether they are one- or two-sided<br>*Only common tests should be described solely by name; describe more complex techniques in the Methods section.*
☒	☐	A description of all covariates tested
☒	☐	A description of any assumptions or corrections, such as tests of normality and adjustment for multiple comparisons
☐	☒	A full description of the statistical parameters including central tendency (e.g. means) or other basic estimates (e.g. regression coefficient) AND variation (e.g. standard deviation) or associated estimates of uncertainty (e.g. confidence intervals)
☒	☐	For null hypothesis testing, the test statistic (e.g. *F*, *t*, *r*) with confidence intervals, effect sizes, degrees of freedom and *P* value noted<br>*Give P values as exact values whenever suitable.*
☒	☐	For Bayesian analysis, information on the choice of priors and Markov chain Monte Carlo settings
☒	☐	For hierarchical and complex designs, identification of the appropriate level for tests and full reporting of outcomes
☒	☐	Estimates of effect sizes (e.g. Cohen's *d*, Pearson's *r*), indicating how they were calculated

Our web collection on statistics for biologists contains articles on many of the points above.

Software and code

Policy information about availability of computer code

Data collection	No software was used.
Data analysis	QSR's NVivo 11 qualitative data analysis software (QSR International Pty Ltd., 2017)

For manuscripts utilizing custom algorithms or software that are central to the research but not yet described in published literature, software must be made available to editors and reviewers. We strongly encourage code deposition in a community repository (e.g. GitHub). See the Nature Research guidelines for submitting code & software for further information.

Data

Policy information about availability of data

All manuscripts must include a data availability statement. This statement should provide the following information, where applicable:

- Accession codes, unique identifiers, or web links for publicly available datasets
- A list of figures that have associated raw data
- A description of any restrictions on data availability

The data that support the findings of this study are available from the corresponding author upon reasonable request.

Field-specific reporting

Please select the one below that is the best fit for your research. If you are not sure, read the appropriate sections before making your selection.

☐ Life sciences ☒ Behavioural & social sciences ☐ Ecological, evolutionary & environmental sciences

For a reference copy of the document with all sections, see nature.com/documents/nr-reporting-summary-flat.pdf

Behavioural & social sciences study design

All studies must disclose on these points even when the disclosure is negative.

Study description	Mixed-methods
Research sample	Adults (aged 18 years or over) with asthma were recruited to explore user engagement with a digital self-management intervention for asthma, and identify factors that may influence engagement. Mean age across samples ranged from 56.8-60.3 years and percentage of females ranged from 61.4-66.7%.
Sampling strategy	Participants were recruited from the intervention arm of a feasibility randomised controlled trial. Eligible participants were identified and invited to take part in the feasibility trial by seven general practices from the Wessex, UK primary care research network. All intervention group participants (n=44) were invited to take part in a qualitative interview. Drawing on the guidelines on information power in qualitative interview studies, we aimed to recruit approximately 20 participants to the interview study. This number was deemed adequate given the study's narrow aim (views on one intervention), the small source population (n=44), the specificity of the experiences, knowledge and properties among the intervention trial participants, and the likely high quality of dialogue from using an experienced qualitative researcher. For the qualitative interviews, data saturation was considered reached because participants in later interviews did not indicate any significant new benefits, concerns or barriers to engagement with My Breathing Matters.
Data collection	Quantitative usage data (automatically by the intervention software) were collected to describe patterns of intervention usage over the 12-month study period. The My Breathing Matters Satisfaction Questionnaire was administered to intervention participants at 12-month follow-up to assess their satisfaction with the intervention. Qualitative semi-structured telephone interviews were carried out to explore intervention participants' views and experiences of My Breathing Matters.
Timing	Trial recruitment began in March 2017 and was completed in August 2017. Quantitative data collection ended in August 2018. Interviews took place between July 2017 and January 2018.
Data exclusions	No data were excluded from the analysis.
Non-participation	Participants who did not take part in the interview either withdrew before their interview was due (n=4; 9%), could not be contacted by phone or email after multiple attempts (n=18; 41%) or were too busy (n=4; 9%).
Randomization	Not relevant to this study

Reporting for specific materials, systems and methods

We require information from authors about some types of materials, experimental systems and methods used in many studies. Here, indicate whether each material, system or method listed is relevant to your study. If you are not sure if a list item applies to your research, read the appropriate section before selecting a response.

Materials & experimental systems

n/a	Involved in the study
☒	☐ Antibodies
☒	☐ Eukaryotic cell lines
☒	☐ Palaeontology and archaeology
☒	☐ Animals and other organisms
☐	☐ Human research participants
☒	☐ Clinical data
☒	☐ Dual use research of concern

Methods

n/a	Involved in the study
☒	☐ ChIP-seq
☒	☐ Flow cytometry
☒	☐ MRI-based neuroimaging

Human research participants

Policy information about studies involving human research participants

Population characteristics	See above
Recruitment	See above

Ethics oversight	Ethical approval was granted by the University of Southampton and South Central – Berkshire Research Ethics Committee (REC reference: 16/SC/0614)

Note that full information on the approval of the study protocol must also be provided in the manuscript.
